# Densification and Surface Carbon Transformation of Diamond Powders under High Pressure and High Temperature

**DOI:** 10.3390/ma17030603

**Published:** 2024-01-26

**Authors:** Rongqi Mao, Xiwei Cui, Jinglin Hao, Sizhuang Zhao, Shuai Hou, Fuli Lan, Yanbiao Li, Lifen Deng, He Li

**Affiliations:** 1 College of Mechanical Engineering, Zhejiang University of Technology, Hangzhou 310014, China; maorongqi@nimte.ac.cn (R.M.); lybrory@zjut.edu.cn (Y.L.); 2 Ningbo Institute of Materials Technology and Engineering, Chinese Academy of Sciences, Ningbo 315201, China; cuixiwei@nimte.ac.cn (X.C.); haojinglin@nimte.ac.cn (J.H.); zhaosizhuang@nimte.ac.cn (S.Z.); houshuai@nimte.ac.cn (S.H.); lanfuli@nimte.ac.cn (F.L.); 3 Qianwan Institute of CNITECH, Zhongchuang 1st Road, Zhongchuang Park, Qianwan New Area, Ningbo 315336, China; 4 Center of Materials Science and Optoelectronics Engineering, University of Chinese Academy of Sciences, Beijing 100049, China

**Keywords:** high pressure and high temperature, diamond particle size, densification, Raman spectroscopy, polycrystalline diamonds

## Abstract

A new type of poly-diamond plate without a catalyst was produced via the high-pressure high-temperature (HPHT) compression of diamond powders. The densification of diamond powders and sp^3^ to sp^2^ carbon on the surface under HPHT compression was investigated through the characterization of the microstructure, Raman spectroscopy analysis and electrical resistance measurement. The densification and sp^3^-sp^2^ transformation on the surface are mainly affected by the pressure, temperature and particle size. The quantitative analysis of the diamond sp^3^ and sp^2^ carbon amount was performed through the peak fitting of Raman spectra. It was found that finer diamond particles under a higher temperature and a lower pressure tend to produce more sp^2^ carbon; otherwise, they produce less. In addition, it is interesting to note that the local residual stresses measured using Raman spectra increase with the diamond particle size. The suspected reason is that the increased particle size reduces the number of contact points, resulting in a higher localized pressure at each contact point. The hypothesis was supported by finite element calculation. This study provides detailed and quantitative data about the densification of diamond powders and sp^3^ to sp^2^ transformation on the surface under HPHT treatment, which is valuable for the sintering of polycrystalline diamonds (PCDs) and the HPHT treatment of diamonds.

## 1. Introduction

Polycrystalline diamonds (PCDs) are sintered from micro- or sub-microdiamond powders with the catalysis of transition metals, usually Fe, Ni or Co, via HPHT compression [1,2]. PCDs have been popularly used as abrasion materials and thermal conductors due to their ultra-high wear resistance and excellent thermal conductivity [3]. The polycrystalline diamond compact (PDC), a composite of the PCD and the WC/Co substrate, is produced through the infiltration of Co from the substrate into diamond micropowders as a catalyst via HPHT compression [4]. PDCs combine the PCD’s ultra-high wear resistance and the relatively high impact toughness of the WC/Co substrate [5,6,7]. Consequently, PDCs have been widely employed as machining tools [8], oil and gas drilling bits [9], and grinding wheel dressing tools [10]. The performance of PDCs is generally believed to increase with the diamond content; therefore, it is of primary importance to improve the diamond density by optimizing the particle size distribution. The sintering of PDCs includes three main processes [11]: (1) cold compaction—where diamond powders are densified via fragmentation and rearrangement with the pressure increasing from the atmosphere to a high pressure of up to 7 or 8 GPa; (2) hot pressing—as the temperature increases to above 800 °C, diamond particles could be elastically deformed and the powders are hot compacted to become denser; (3) cobalt melting and infiltration—the catalyst cobalt infiltrates into diamond powders and assists in the formation of diamond to diamond bonds. The literature [12,13,14] mainly focuses on the third process, the influence of the synthesis pressure and temperature on PDC sintering. However, there is little research on the first and second process. Our group has conducted a detailed study on cold compaction and found that the packing density of diamond powder with two different particle sizes is higher than the one with a single particle size. For example, the density of the 2–4 μm:20–30 μm (1:3) powder increases by 27% to 50% after cold pressing compared to the density of the 2–4 μm powder [15]. Following that work, this paper investigated the further densification during the hot pressing. Before liquid catalyst cobalt infiltration, diamond particle surfaces experience different pressures depending on their contacting conditions. The actual pressure applied on the diamond surface around a space would be particularly lower than that on those in contact with each other, resulting in a partial sp^3^-sp^2^ transformation at high temperatures. The sp^2^ carbon would affect the following diamond sintering. With the increase in sp^2^ carbon, the hardness of the sintered PDCs will decrease. In order to further develop the properties of PCDs and PDCs, this paper also studied the effects of the pressure, heating power and particle size on the sp^3^ to sp^2^ carbon transformation on surfaces. 

In addition, although there are extensive studies on the transformations of diamond into graphite at high temperatures, including natural single crystalline diamonds, HPHT and Chemical Vapor Deposition (CVD) synthesized diamonds and diamond composites [16,17,18,19,20,21], with the majority of the studies conducted at atmosphere, there is little research on diamond graphitization at high pressures. G. Davies [22] measured the graphitization of diamonds under high-pressure and zero-pressure conditions and calculated the activation energy of graphitization. Qian, J. [23] conducted high-temperature and high-pressure treatment on nanoscale diamonds and characterized the graphitization. The present study systematically investigated the densification and sp^3^ to sp^2^ transformation on the surface of microdiamond powders after high-pressure hot pressing, which is useful for the synthesis optimization of polycrystalline diamonds.

On the other side, the synthesis of polycrystalline diamonds via CVD usually employs substrates such as silicon and molybdenum [24,25]. Due to the thermal expansion coefficient difference between the diamond and substrate, huge stresses are introduced and could result in the cracking of CVD polycrystalline diamonds. In order to address this problem, a new type of substrate with a similar thermal expansion coefficient is desirable. The PCDs sintered via HPHT treatment have been used for CVD substrates, but they did not work due to the existence of catalyst cobalt. In this study, a new type of poly-diamond plate without cobalt has been produced. It has enough strength for a CVD substrate and possesses a thermal expansion coefficient similar to CVD polycrystalline diamonds. This hot-compressed poly-diamond plate shows promise in decreasing thermal stresses during the CVD synthesis of polycrystalline diamonds when used as a substrate. 

## 2. Experimental Procedures

### 2.1. Materials

The diamond powders used in this experiment were provided by Henan Union Precision Share Co., Ltd., Zhengzhou, China, with the brand name of HDP-PCD. Five diamond powders with particle size ranges of 2–4 μm (G_2–4_), 6–12 μm (G_6–12_), 8–16 μm (G_8–16_), 15–25 μm (G_15–25_) and 20–30 μm (G_20–30_) were used and named as samples A, B, C, D and E.

### 2.2. HPHT Experiment

The hot-pressing experiments were conducted using a Φ800 cubic press YMTS800, which is produced by Zhengzhou Abrasives Grinding Research Institute Co., Ltd., Zhengzhou, China. Before hot pressing, different particle sizes of diamond powders with the same mass were weighed using an electronic balance. These five diamond powder samples were individually placed into niobium cups and then loaded into the same pyrophyllite cube to ensure that the temperature and pressure of different particle sizes of diamond powders were kept consistent during the hot-pressing process. The experiment used two different pressures (30 MPa and 40 MPa) and three different temperatures (4 kW (1023 °C), 5 kW (1256 °C) and 6 kW (1494 °C)). After a 5 min hot-pressing process, the pyrophyllite cube was allowed to cool naturally before being removed from the cubic press, then the pyrophyllite cubes were knocked open and hot-pressed samples were taken out. 

### 2.3. Characterization Methods

After breaking these plate samples, the microscopic microstructures of the fracture surfaces were observed using a scanning electron microscope (SEM) that was produced by Germany Zeiss (Jena, Germany). The instrument model was G300, and the voltage of the instrument was 0.2–20 kV, which can be adjusted continuously in steps of 10 V. In addition, the secondary imaging resolution was 0.7 nm; the magnification of the image was 12~2,000,000 times. In this paper, a voltage of 15 kV was selected. The density was measured using the drainage method. All samples’ XRD spectra were measured, but the results did not show a noticeable graphite peak. Therefore, the sp^2^ carbon on surfaces of diamond powders after hot pressing was analyzed using France Horiba Raman spectroscopy. The Raman spectrometer has a resolution of 0.65 cm^−1^ and power of 100 mW/μm^2^. The displacement range is 50 cm^−1^ to 9000 cm^−1^. The displacement range used in this study is 200 cm^−1^ to 3000 cm^−1^, and the Raman spectrometer has four laser wavelengths, which are 325 nm, 532 nm, 633 nm and 785 nm. The instrument model was the LabRAM Odyssey. The electrical resistance of hot-pressed diamond samples with different components were measured via direct measurement using an Omron multimeter using the average value of 5 measurements. All samples prepared under various experimental conditions are listed in Table S1. 

## 3. Results

### 3.1. Diamond Particle Size and Hot-Pressed Conditions

**Figure 1:**
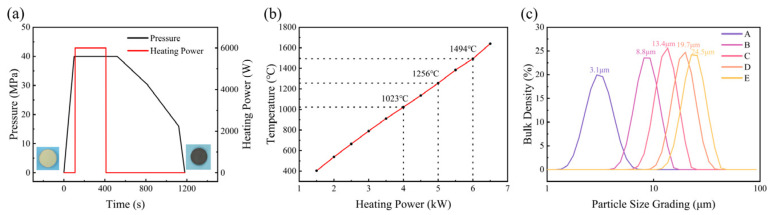
(**a**) Hot-pressed conditions; (**b**) capsule temperature–heating power curve; (**c**) diamond particle size distributions.

Figure 1a shows the pressure P-t and heating power curves that were used to hot press diamond powders into a compacted plate sample. The function of the pressure change with time is P = 0.475 t, and the function of the temperature change with time is T = 7.3 t. The actual heating temperatures corresponding to various heating powers were measured using a platinum–rhodium thermocouple, and the results are shown in Figure 1b. The actual pressure inside the cavity is calibrated through the phase transition of bismuth and barium under high pressure, and the corresponding relationship between system pressure and actual pressure inside the cavity is obtained [26]. The actual pressures in the sample capsule for 30 MPa and 40 MPa hydraulic pressures are estimated to be around 5 GPa and 7.7 GPa, respectively. The particle size distribution of five diamond powder samples were measured using a micron laser particle size analyzer, the Malvern MS 300, and the results are shown in Figure 1c. The average particle sizes corresponding to samples A, B, C, D and E are 3.1 μm, 8.8 μm, 13.4 μm, 19.7 μm and 24.5 μm, respectively.

### 3.2. Densities and Microstructure of Hot-Pressed Diamond Samples

**Figure 2:**
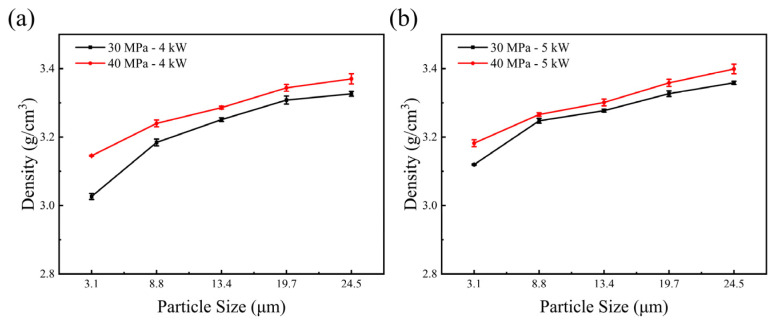
(**a**) Densities of diamond samples A, B, C, D and E after hot pressing under 30 MPa-4 kW and 40 MPa-4 kW; (**b**) densities of diamond samples A, B, C, D and E after hot pressing under 30 MPa-5 kW and 40 MPa-5 kW.

The density of diamond samples with different particle sizes after hot pressing at 30 MPa-4 kW and 40 MPa-4 kW are shown in Figure 2. With increases in the diamond particle size, the density of the hot-pressed diamond increases and the porosity decreases. Sample E diamonds became remarkably dense after the treatments at 40 MPa-4 kW and reached 3.4 g/cm^3^, which is 97.1% of the theoretical density (3.5 g/cm^3^) of the diamond, which means that there is only 2.9 vol% porosity in the sample. For a given heating power, the samples became denser as the pressure increased. For a given pressure, the density slightly increased as the heating power inclined from 4 kW to 5 kW, which indicates that diamond elastically deformed more at a higher temperature. Due to the difficulty of removing the outside niobium cup, the densities of 6 kW samples have not been measured. 

**Figure 3:**
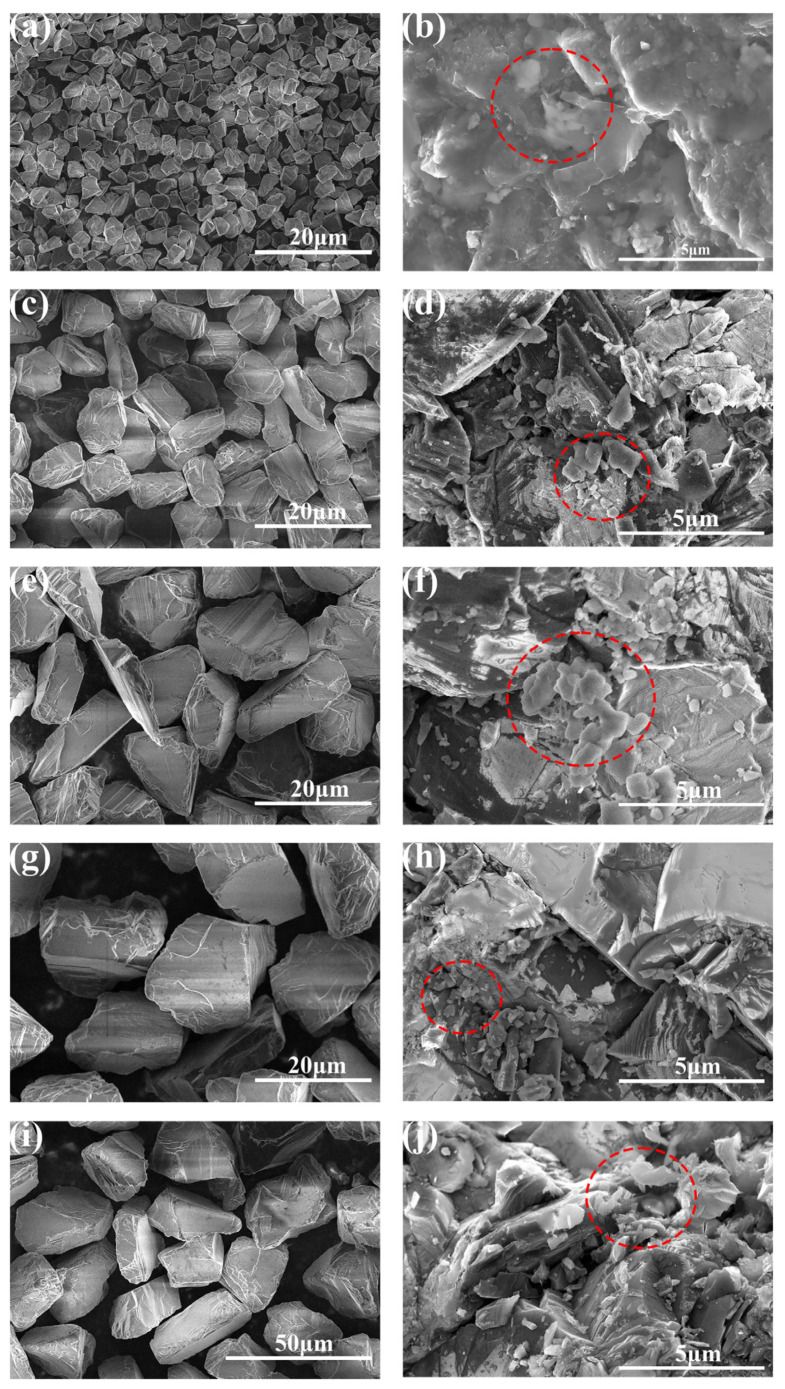
SEM images of 30 MPa and 6 kW samples A, B, C, D and E: (**a**,**c**,**e**,**g**,**i**) are samples A, B, C, D and E before being hot pressed, respectively; (**b**,**d**,**f**,**h**,**j**) are samples A, B, C, D and E after being hot pressed, respectively (The red dashed circle represents diamond particles that become smooth and rounded after HPHT).

Figure 3 shows the microstructures of sample A, sample B, sample C, sample D and sample E before and after 30 MPa and 6 kW hot pressing. Figure 3a,c,e,g,i are images before hot pressing, and it can be seen that the diamond particles retain their individual shapes, and their size gradually increases. Figure 3b,d,f,h,j are images after hot pressing. It is observed that the features of the cleavage surface on the original diamond particles have disappeared. The sharp edges on the surface have become smooth and rounded, and the particles are densely packed, as shown in Figure 3e,f. Furthermore, diamond particles can be observed bonding together in certain areas, and it is evident that diamond particles of various sizes have experienced crushing during HPHT treatment, with the smaller crushed particles filling the spaces around the larger particles. These smaller diamond particles have transformed into sheet-like structures after HTHP treatment. This indicates significant graphitization occurred during hot pressing. This may be due to the pressure between the large diamond grains being relatively low, resulting in the graphitization of the smaller crushed particles. Although some larger-sized components have experienced partial crushing during HPHT treatment, the percentage of fine particles is still the highest in sample A and the lowest in sample E.

### 3.3. Electrical Resistance of Hot-Pressed Samples

**Figure 4:**
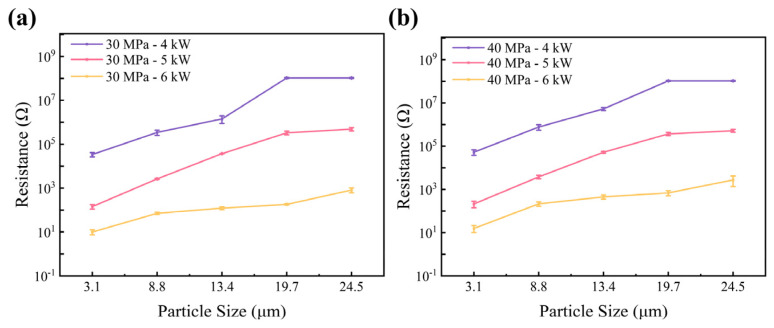
Electrical resistance of the 30 MPa and 40 MPa hot-pressed diamond samples: (**a**) electrical resistance of hot-pressed diamond samples with different particle sizes under 30 MPa; (**b**) electrical resistance of hot-pressed diamond samples with different particle sizes under 40MPa.

The surfaces of the diamond samples after hot pressing were measured using an Omron multimeter. The electrical resistance was measured using the average value of five measurements. The results indicate that the HPHT hot-pressed diamond samples exhibit a certain level of electrical conductivity. In general, the pure diamond powder is non-conductive, while Figure 4a,b shows the electrical resistance of five samples hot pressed at 30 MPa and 40 MPa hydraulic pressures. It is evident that as the diamond particle size increases, the electric resistance of hot-pressed diamond samples gradually increases. This indicates that under the same treatment, smaller diamond particles (G_2–4_) have more sp^3^ carbon transformed to sp^2^ or amorphous carbon compared to coarser particles (G_20–30_), resulting in a reduction in the electrical resistance. This may be due to the higher pressure exerted on coarser diamond particles (G_20–30_) compared to smaller diamond particles (G_2–4_), resulting in the sp^3^ carbon on smaller diamond particles’ surfaces being less stable. It can be seen in Figure 4 that as the power increases, the electrical resistance decreases. This validates that at the same pressure, a higher temperature led to a higher degree of surface sp^3^ to sp^2^ carbon transformation. 

### 3.4. Raman Spectra

Raman spectroscopy is an inelastic light scattering process that allows for the identification and characterization of the structure of molecules from gas to solid phases and from amorphous to crystals. In material sciences, it is commonly used to characterize the molecular structures of carbon-based materials and associated defects [27]. The Raman peaks of diamond and graphite are located at different positions, with 1332 cm^−1^ being the diamond peak and 1580 cm^−1^ being the graphite peak. Figure S1 shows the Raman and PL spectra of the original diamond. This indicates that nitrogen does exist in the original diamond. Figure S2 shows the Raman spectra of diamond samples with three different wavelengths of lasers: 325 nm, 532 nm and 785 nm. From the graph, it can be seen that the 1332 cm^−1^ peak is the diamond peak.

**Figure 5:**
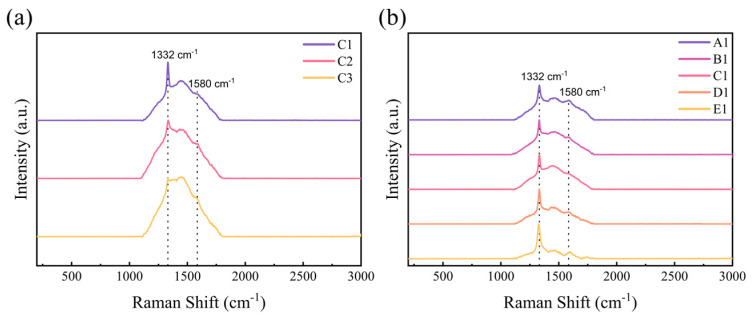
(**a**) Raman spectra of sample C after the treatments at 30 MPa with various heating powers; (**b**) Raman spectra of the 30 MPa-4 kW hot-pressed diamond samples A, B, C, D and E.

After removing the baseline through polynomial fitting and normalizing the diamond peaks of each sample, the results are shown in Figure 5. 

From Figure 5a, it can be observed that for sample C pressed at 30 MPa, as the heating temperature increases, the G peak at 1580 cm^−1^ gradually rises, as well as the amorphous peak in the range of 1450 cm^−1^ to 1555 cm^−1^. Figure 5b showed that for samples treated with 4 kW power and 30 MPa pressure, as the average particle size increases, the G peak at 1580 cm^−1^ gradually decreases, and the amorphous peak in the range of 1450 cm^−1^ to 1555 cm^−1^ also decreases. 

During the hot-pressing stage, various carbon substances with sp^2^ and sp^2^/sp^3^ hybridization of carbon can be formed. From Figure 5, we did not see the sp^2^ peak at the low-frequency spectrum [28] or the second-order Raman peak [29] due to the insufficient sensitivity of the device. The peak shape is concentrated between 1000 cm^−1^ and 2000 cm^−1^. To quantitatively analyze the influence of pressure and temperature on the surface carbon phase transformation, select Raman spectra in the range of 800–2000 cm^−1^ for seven-peak fitting using Origin 2021 software. According to the references [30,31], the peak positions obtained through Gaussian fitting and their possible origins are listed in Table 1, and the peaks are numbered from left to right. The R^2^ values for all spectra during fitting are at least 0.995.

**Figure 6:**
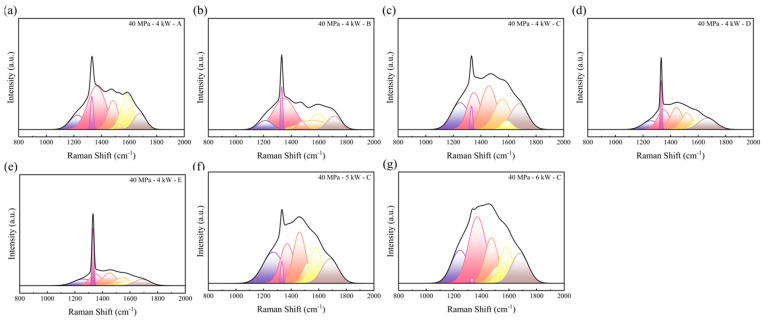
Diamond Raman fitting peak: (**a**) sample A with 40 MPa-4 kW; (**b**) sample B with 40 MPa-4 kW; (**c**) sample C with 40 MPa-4 kW; (**d**) sample D with 40 MPa-6 kW; (**e**) sample E with 40 MPa-6 kW; (**f**) sample C with 40 MPa-5 kW; (**g**) sample C with 40 MPa-6 kW.

Figure 6a–e represent hot-pressed samples of components A, B, C, D and E from 40 MPa to 4 kW, respectively. Figure 6c,f,g are 40 MPa -powder C samples treated with 4 kW, 5 kW and 6 kW heating powers. Other components can be found in Figure S4. 

**Figure 7:**
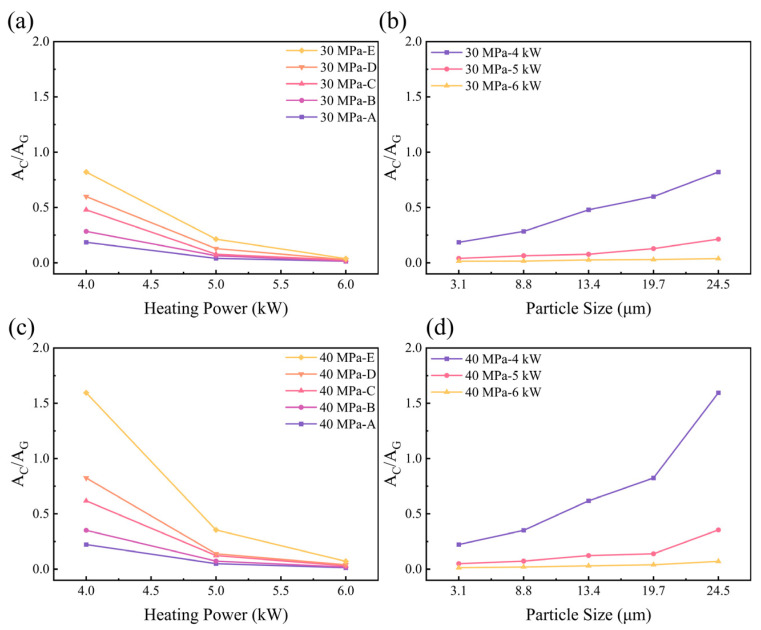
Diamond samples’ graphite content parameter: (**a**) 30 MPa with different particle sizes; (**b**) 30 MPa with different heating powers; (**c**) 40 MPa with different particle sizes; (**d**) 40 MPa with different heating powers.

The sharp peak at 1330 cm^−1^ to 1340 cm^−1^ is characteristic of the first-order Raman line of crystalline diamond [33,34,35], and it indicates the presence of the diamond in the sample. As the heating power increases, the diamond peak area gradually decreases. The peak at 1575 cm^−1^ to 1600 cm^−1^ is caused by the “G peak” of sp^2^ carbon [36,37]; it indicates the conversion of the surface diamond into graphite after HPHT treatment. As the heating power increases, the G peak area gradually increases. The graphite content is represented by the ratio of the diamond peak area to the graphite G peak area (A_C_/A_G_) [38,39,40,41], as shown in Figure 7.

From Figure 7, it can be seen that under the same heating power and pressure conditions, as the diamond particle size increases, the A_C_/A_G_ gradually increases, which indicates a lower degree of graphitization. For the same diamond particle size and pressure conditions, as the heating power increases, the A_C_/A_G_ gradually decreases, which indicates a higher degree of graphitization.

### 3.5. Influence of Particle Sizes and Heating Powers on the Residual Stresses in Hot-Pressed Samples

A Raman spectrometer was employed to measure the diamond peak shifts of various diamond samples and calculate the residual stresses. In order to remove the particle size effect on the Raman spectra diamond peak [42], the reference peak position for five diamond powders with various particle sizes were determined individually using the average value of 10 measurements. The residual stresses were calculated using the Raman peak shifts after HTHP treatments. The results are shown in Table 2.

The stress values were calculated using the following formula [43,44]:

(1) $$\sigma_{b}=\frac{\gamma_{0}-\gamma }{2.88}$$

where σ_b_ is the residual stress, γ_0_ is the reference peak and γ is the diamond peak. Negative values represent compressive stress, while positive values indicate tensile stress. As seen in Table 2, all measured samples exhibited residual compressive stress. The residual compressive stress increases with increases in the particle size and also increases with higher heating temperatures. For the same diamond powders, as the pressure increases from 30 MPa to 40 MPa, the residual stress also increases.

**Figure 8:**
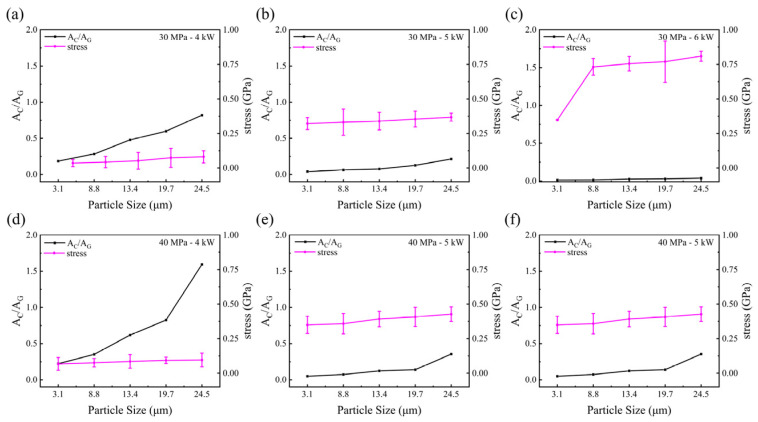
The graphite content parameter (A_C_/A_G_) and the residual stresses of hot-pressed samples vary with particle sizes: (**a**) 30 MPa-4 kW; (**b**) 30 MPa-5 kW; (**c**) 30 MPa-6 kW; (**d**) 40 MPa-4 kW; (**e**) 40 MPa-5 kW; (**f**) 40 MPa-6 kW.

Figure 8 shows the A_C_/A_G_ and residual stress of hot-pressed samples plotted against the particle size. All data indicated that the residual internal stresses increased with increases in the particle size, although the trends were different as the HPHT parameters changed. As the diamond particle size increased, the increased residual stresses further proved that larger diamond particle surfaces have experienced higher local stresses. As a result, the graphite content decreased correspondingly.

**Figure 9:**
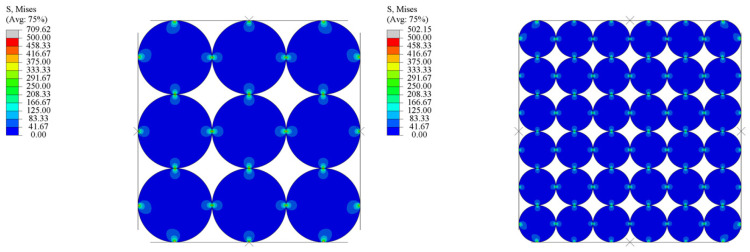
Finite element simulation of stresses on diamond particles.

To deeply delve into the impact of the diamond particle size on residual internal stress, a two-dimensional finite element model was established using the finite element simulation software Abaqus 2020. Ignoring the irregular shapes of diamond particles, the diamond particles were represented as circular shapes. Force analyses were simulated for 7.5 mm and 30 mm diamond particles under identical external pressures. Figure 9 depicts the simulation results when the same external force was applied. The deeper the color, the higher the stress.

It clearly demonstrates that larger particles experience a higher pressure under the same external forces compared to smaller ones. The reason is that the increase in particle size leads to a reduction in the number of contact points, resulting in a higher localized pressure at each contact point, and the pressure is relatively low in the non-contact position. This explains why the residual stress increases with increases in the diamond powder particle size under the same temperature and pressure conditions. Furthermore, we believe that a higher pressure on the diamond surface is more conducive to maintaining the sp^3^ structure, which provides an explanation for the decrease in the graphite content as the diamond powder particle size increases. In additions, it is interesting to note that the residual stresses incline with increases in the heating temperature. This is because diamond particles deform more and reach a denser compact at a higher temperature; when they cool down, more residual stresses are left on the diamond particles’ surface.

## 4. Conclusions and Prospects

In summary, a series of poly-diamond plates were produced using a cubic press with a range of 6–8 GPa and 1000–1500 °C using various sizes of microdiamond powders. Some phenomena were found as follows:

(1) After HPHT treatment, the diamond particles were observed to bond together in certain areas, resulting in the formation of diamond plate samples exhibiting both strength and some electrical conductivity.
(2) During the HPHT hot-compressing stage, diamond powders were further densified via the elastic and non-elastic deformation of diamond powders, and the density can reach up to 3.4 g/cm^3^, 97.1% of the diamond itself, approaching a full dense diamond plate. The density increases with the diamond particle size, pressure and temperature.
(3) Diamond powders with different particle sizes undergo varying degrees of sp^3^ to sp^2^ or amorphous carbon transformation on the surface. The graphite content on diamond surfaces increases as the particle size decreases. There are two primary reasons for this phenomenon. Firstly, smaller particles have a higher surface energy, resulting in a lower activation energy for graphite transformation and making the diamond more prone to graphitize. Secondly, smaller particles possess a higher number of contact areas, which can reduce the local stress on the diamond under the same pressure. Conversely, coarser diamond particles have a lower surface energy, higher activation energy for graphite and fewer contact areas. This can result in higher residual stresses and a reduced graphitization under the same pressure.

These valuable data are important to understand the hot-press stage of diamond powders, which could facilitate the optimization of the HPHT sintering process of PCDs and the HPHT treatment of diamonds. Due to the inherent low strength and similar thermal expansion coefficients of these hot-pressed diamond plates, these hot-pressed samples could be suitable as CVD polycrystalline diamonds’ substrates. Furthermore, with an adjustable electrical conductivity combined with a microporous microstructure and diamonds’ high resistance to chemicals, the poly-diamond plates might find some potential application in the electrical chemistry industry as electrodes.

## Figures and Tables

**Table 1:** Seven peaks’ positions and boundaries.

Peak Number	Source of Characteristic Peaks [27,32]	Characteristic Peak Boundary (cm^−1^)
D4	Carbon black and multi walled carbon nanotubes	1210~1290
C	Diamond	1330~1340
D1	Lattice vibration of disordered graphite	1350~1390
D3′	Amorphous	1450~1490
D3″	Amorphous	1520~1555
G	Ordered graphitization	1575~1590
D2/D′	Defects due to the breaking of the crystal symmetry	1615~1650

**Table 2:** Raman diamond peaks and residual stresses of hot-pressed samples.

Hydraulic Pressure (MPa)	Power (kW)	Particle Size (μm)	Sample	Diamond Peak (cm^−1^)	Reference Peak (cm^−1^)	Peak Shift (cm^−1^)	Stress (GPa)
30 MPa	4 kW	G_2–4_	A1	1332.01	1331.91	−0.10	−0.04
		G_6–12_	B1	1332.11	1331.99	−0.12	−0.04
		G_8–16_	C1	1332.16	1332.01	−0.15	−0.05
		G_15–25_	D1	1332.29	1332.08	−0.21	−0.07
		G_20–30_	E1	1332.36	1332.13	−0.23	−0.08
40 MPa	4 kW	G_2–4_	A4	1332.10	1331.91	−0.19	−0.07
		G_6–12_	B4	1332.21	1331.99	−0.22	−0.08
		G_8–16_	C4	1332.26	1332.01	−0.25	−0.09
		G_15–25_	D4	1332.35	1332.08	−0.27	−0.09
		G_20–30_	E4	1332.46	1332.13	−0.33	−0.12
30 MPa	5 kW	G_2–4_	A2	1332.84	1331.91	−0.93	−0.32
		G_6–12_	B2	1332.94	1331.99	−0.95	-0.33
		G_8–16_	C2	1332.99	1332.01	−0.98	−0.34
		G_15–25_	D2	1333.10	1332.08	−1.02	−0.35
		G_20–30_	E2	1333.19	1332.13	−1.06	−0.37
40 MPa	5 kW	G_2–4_	A5	1332.92	1331.91	−1.01	−0.35
		G_6–12_	B5	1333.02	1331.99	−1.03	−0.36
		G_8–16_	C5	1333.14	1332.01	−1.13	−0.39
		G_15–25_	D5	1333.25	1332.08	−1.17	−0.41
		G_20–30_	E5	1333.36	1332.13	−1.23	−0.43
30 MPa	6 kW	G_2–4_	A3	1334.00	1331.91	−2.09	−0.73
		G_6–12_	B3	1334.10	1331.99	−2.11	−0.73
		G_8–16_	C3	1334.18	1332.01	−2.17	−0.75
		G_15–25_	D3	1334.30	1332.08	−2.22	−0.77
		G_20–30_	E3	1334.46	1332.13	−2.33	−0.81
40 MPa	6 kW	G_2–4_	A6	1334.05	1331.91	−2.14	−0.74
		G_6–12_	B6	1334.17	1331.99	−2.17	−0.76
		G_8–16_	C6	1334.25	1332.01	−2.24	−0.78
		G_15–25_	D6	1334.38	1332.08	−2.30	−0.80
		G_20–30_	E6	1334.50	1332.13	−2.37	−0.82

## Data Availability

Data are contained within the article.

## Supplementary Materials

The following supporting information can be downloaded at https://www.mdpi.com/article/10.3390/ma17030603/s1, Figure S1: (a) Original diamond Raman spectroscopy and (b) original diamond PL spectrum; Figure S2: Raman spectra of hot-pressed diamonds with three different wavelengths; Figure S3: Raman spectra of each hot-pressed diamond sample; Figure S4: Fitted Raman spectra of each hot-pressed diamond sample; Table S1: Experimental conditions for hot-pressed samples.
