# Supplementary material for: Densification and Surface Carbon Transformation of Diamond Powders under High Pressure and High Temperature
Source: Materials (Basel). 2024 Jan 26;17(3):603. doi: 10.3390/ma17030603 (PMC10856571; doi:10.3390/ma17030603)
Supplement: Supplementary file 1 [file materials-17-00603-s001.zip › materials-2804886-supplementary.pdf]

Supporting information

Densification and surface carbon transformation of diamond powders under High Pressure and High Temperature

Rongqi Mao, Xiwei Cui, Jinglin Hao, Sizhuang Zhao, Shuai Hou, Fuli Lan, Yanbiao Li, Lifen Deng and **He Li**

1. Experimental conditions for hot pressed samples

All samples produced using various experimental conditions lists in Table S1.

Table S1. Experimental conditions for hot pressed samples

Sample	Hydraulic Pressure (MPa)	Cavity pressure (GPa)	Power (kW)	Temperature (℃)	Particle Size (μm)	Average Particle Size (μm)
A1	30 MPa	5.3 GPa	4 kW	1023 ℃	G2-4	3.1
B1					G6-12	8.8
C1					G8-16	13.4
D1					G15-25	19.7
E1					G20-30	24.5
A2			5 kW	1256 ℃	G2-4	3.1
B2					G6-12	8.8
C2					G8-16	13.4
D2					G15-25	19.7
E2					G20-30	24.5
A3			6 kW	1494 ℃	G2-4	3.1
B3					G6-12	8.8
C3					G8-16	13.4
D3					G15-25	19.7
E3					G20-30	24.5
A4	40 MPa	6.8 GPa	4 kW	1023 ℃	G2-4	3.1
B4					G6-12	8.8
C4					G8-16	13.4
D4					G15-25	19.7
E4					G20-30	24.5
A5			5 kW	1256 ℃	G2-4	3.1
B5					G6-12	8.8
C5					G8-16	13.4
D5					G15-25	19.7
E5					G20-30	24.5
A6			6 kW	1494 ℃	G2-4	3.1
B6					G6-12	8.8
C6					G8-16	13.4
D6					G15-25	19.7
E6					G20-30	24.5

2. Raman Spectra

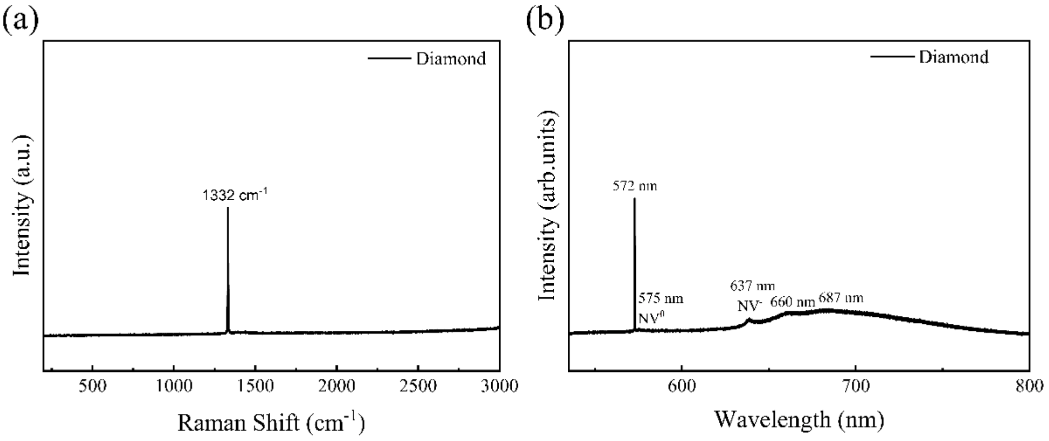

Figure S1. (a) Original diamond Raman Spectroscopy; (b) Original diamond PL spectrum;

Fig. S1 shows the Raman and PL spectra of the original diamond. This indicates that nitrogen does exist in the original diamond.

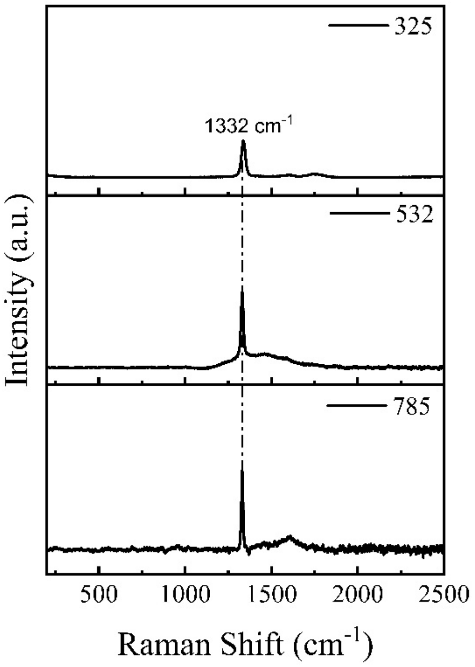

Figure S2. Raman spectra of hot pressed diamond with three different wavelengths;

Fig. S2 shows the Raman spectra of diamond samples with three different wavelengths of lasers: 325nm, 532nm, and 785nm. From the graph, it can be seen that there is a diamond peak at 1332cm-1.

The Raman spectra of hot-pressed samples after normalization of the diamond peak were presented in

Figure S3. Figure S3 (a), (b), (c), (d), (e) are the Raman spectra of hot-pressed samples of 30 MPa - 5 kW, 30 MPa - 6 kW, 40 MPa - 4 kW, 40 MPa - 5 kW, 40 MPa - 6 kW, respectively. Figure S5 (f), (g), (h), (i), (j) (k), (l), (m), (n) are the Raman spectra of hot-pressed samples of 30 MPa - A, 30 MPa - B, 30 MPa - D, 30 MPa - E, 40 MPa - A, 40 MPa - B, 40 MPa - C, 40 MPa - D, 40 MPa - E, respectively.

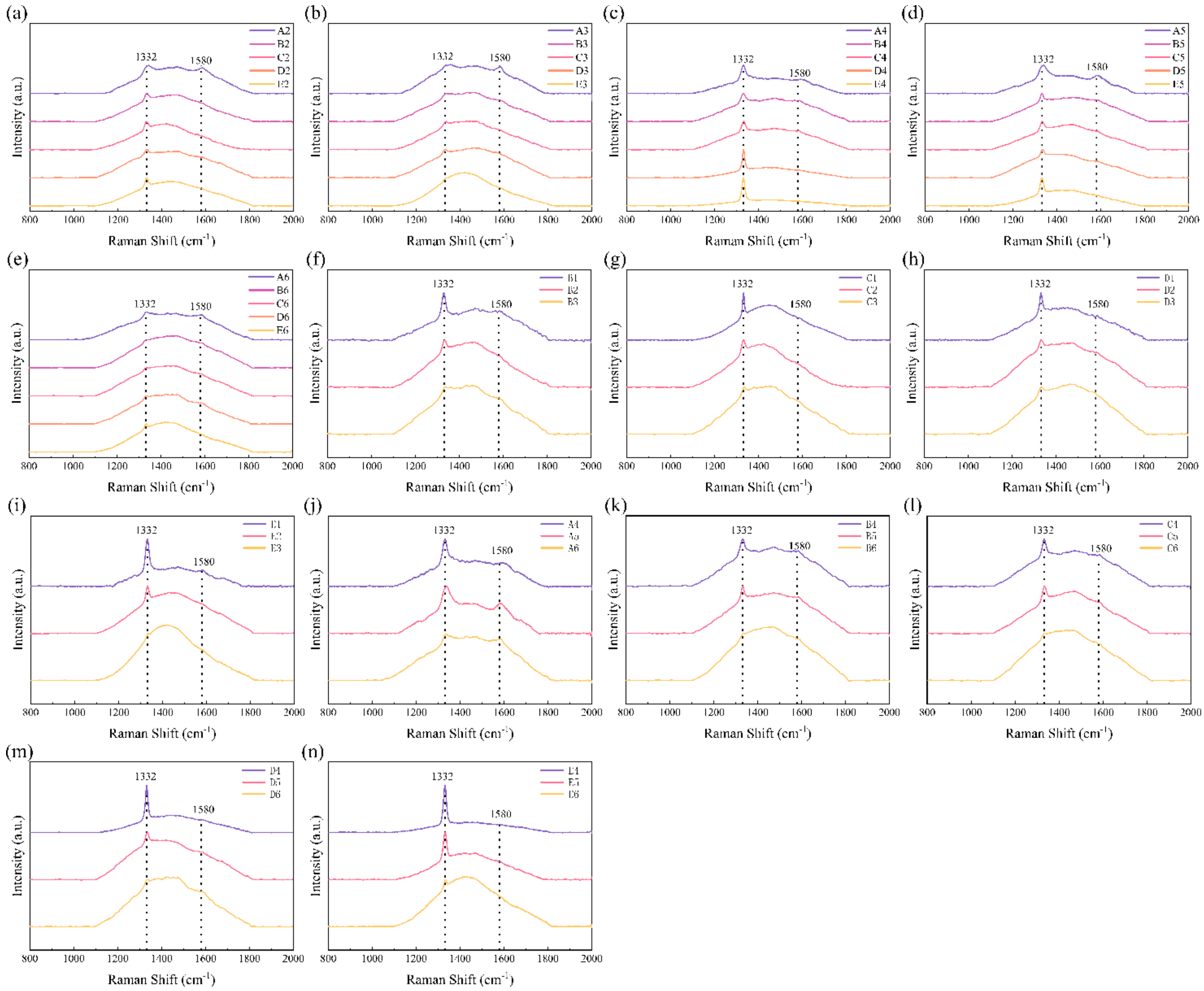

Figure S3. Raman spectra of each hot-pressed diamond samples

Fitted Raman image were shown in Figure S4. Figure S4 (a), (b), (c), (d) and (e) represent hot-pressed samples of components A, B, C, D, E from 30 MPa to 4 kW, respectively. Figure S6 (f), (g), (h), (i) and (j) represent hot-pressed samples of components A, B, C, D, E from 30 MPa to 5 kW, respectively. Figure S4 (k), (l), (m), (n) and (o) represent hot-pressed samples of components A, B, C, D, E from 30 MPa to 6 kW, respectively. Figure S6 (p), (q), (r) and (s) represent hot-pressed samples of components A, B, D, E from 40 MPa to 5 kW, respectively. Figure S4 (t), (u), (v) and (w) represent hot-pressed samples of components A, B, D, E from 40 MPa to 6 kW,

respectively.

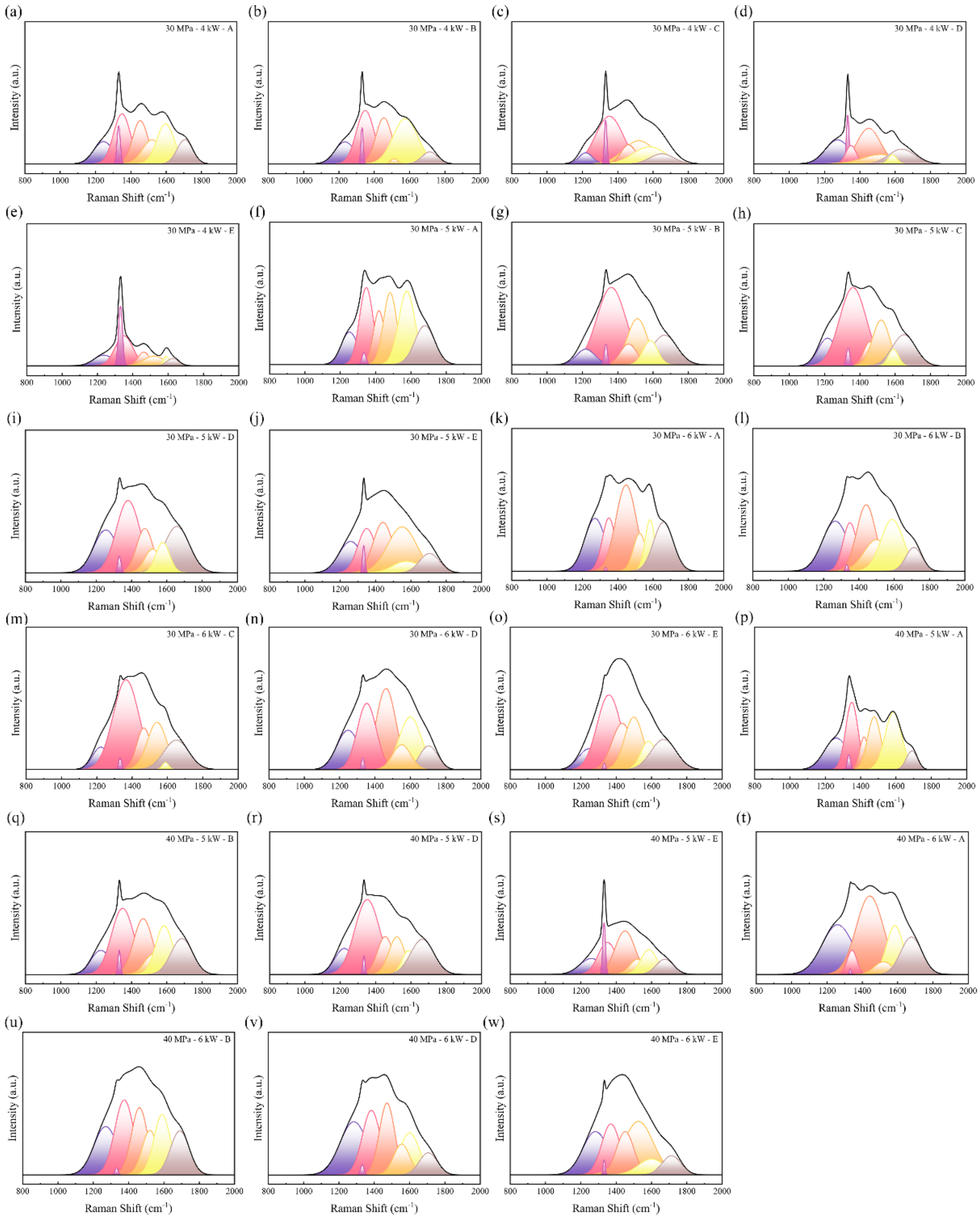

Figure S4. Fitted Raman of each hot-pressed diamond samples
